# A Case of Nasopharyngeal Mycobacteriosis with Bony Erosion of the External Skull Base

**DOI:** 10.1155/2021/7500273

**Published:** 2021-10-15

**Authors:** Kohei Matsuo, Satoshi Tanaka, Masayuki Sakata, Hiroki Takeda, Akihiro Nagata, Masashi Mori, Rie Ito, Yoshifumi Yamamoto, Kiyonobu Ueno, Atsuhiko Uno

**Affiliations:** ^1^Otorhinolaryngology-Head and Neck Surgery, Osaka General Medical Center, Bandai-higashi 3-1-56, Sumiyoshi-ku, Osaka 558-8558, Japan; ^2^Respiratory Medicine, Osaka General Medical Center, Osaka, Japan

## Abstract

Primary nasopharyngeal mycobacteriosis is a rare disease. We present a case in which skull base bone erosion appeared and was alleviated during the course of the treatment. Bone complications occur in osteoarticular mycobacteriosis, but their occurrence in primary nasopharyngeal mycobacteriosis has not been reported. A 77-year-old immunocompromised Asian woman presented with a right occipitotemporal headache. An ulcerative mass covered with a thick yellowish discharge was found in the roof and posterior walls of the right nasopharynx. Because histopathological examination indicated the presence of mycobacterial infection, we began using antituberculosis medication for the treatment because of the possibility of primary nasopharyngeal tuberculosis. However, this was followed by glossopharyngeal and vagus nerve paralysis. Computed tomography (CT) showed a diffuse enhancing mucosal irregularity in the nasopharynx with bony erosion of the external skull base. Deep tissue biopsy was repeated to differentiate it from malignant lesions, and drainage of pus from the right nasopharynx was confirmed. Subsequently, the headache, neurological findings, and the yellowish discharge disappeared, and the bony erosion of the external skull base was alleviated. Surgical intervention should also be considered for drug-resistant mycobacteriosis. We concluded that mycobacteriosis should also be considered apart from carcinoma even if CT shows a diffuse enhancing mucosal irregularity with bone destruction in the nasopharynx.

## 1. Introduction

Nasopharyngeal mycobacteriosis is a rare disease, and the irregular and swollen mucosal lesion requires differentiation from nasopharyngeal carcinoma. In previous reports of nasopharyngeal mycobacteriosis, lesions were usually found to be confined to the nasopharyngeal area without invasion into the surrounding structures such as the skull base [1, 2], whereas nasopharyngeal carcinoma is often associated with bone destruction. Herein, we report a case of primary nasopharyngeal mycobacteriosis that exhibited bony erosion of the external skull base and transient cranial nerve palsy during the course of the treatment. The pathogen in this case was not identified neither as *Mycobacterium tuberculosis* (TB) nor nontuberculous mycobacteria (NTM).

## 2. Case Presentation

A 77-year-old Asian woman presented with a right occipitotemporal headache for 1 week (day 1) but reported no other aural or nasal symptoms. Her medical history included diabetes mellitus, hypertension, rectal cancer, colon cancer, liver cirrhosis, and hepatocellular carcinoma.

**Figure 1:**
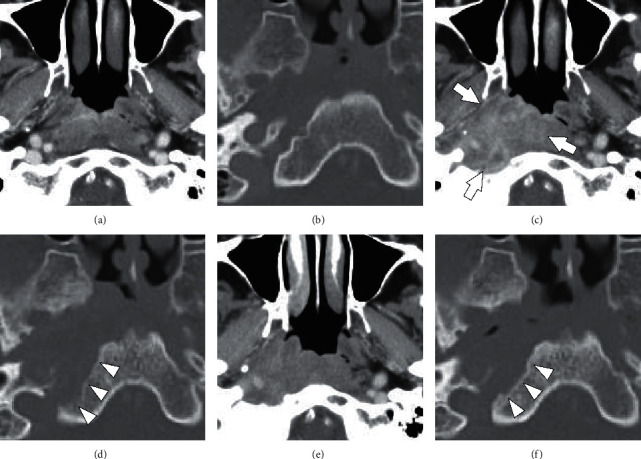
Cervical computed tomography (CT) at the initial visit (a, b) and when glossopharyngeal and vagus nerve paralysis emerged (c, d). CT taken after improvement of symptoms (e, f). The upper panels are contrast-enhanced images of the soft tissue of the nasopharynx (a, c, e). The lower panels are contrast-enhanced images of the bony structure of the extra skull base (b, d, f). The development of diffuse enhancing mucosal irregularity in the nasopharynx (arrow) is shown. (d) The bony erosion of the external skull base (arrow head). (f) The improvement of bony erosion (arrow head).

On physical examination, the right nasopharyngoscopic view revealed an ulcerative mass covered with a thick yellowish discharge that could not be easily removed by suction in the roof and posterior walls of the nasopharynx. However, both otoscopy of the right ear and laryngoscopy showed no abnormalities. No other abnormalities, such as cervical lymphadenopathy, were observed in other head and neck areas. Both the acid-fast *Bacillus* culture test and polymerase chain reaction test of the sputum and nasopharyngeal smear were negative, but the T-SPOT.TB test was positive (both panels A and *B* > 50 spots). Cervical computed tomography (CT) revealed neither a mass shadow in the nasopharynx nor cervical lymphadenopathy (Figure 1(a)). No evidence of bony destruction or intracranial extension was observed (Figure 1(b)). Furthermore, chest and abdominal CT revealed no abnormalities. Histopathological examination showed caseous necrosis in granulomas and epithelioid cell granulomas with Langhans giant cells. Furthermore, Ziehl–Neelsen staining revealed the existence of acid-fast bacilli. Although the differential diagnosis of TB and NTM was a notable challenge, we began using antituberculosis medication for the treatment, consisting of a combination of rifampicin (R, 600 mg), isoniazid (H, 300 mg), ethambutol (E, 750 mg), and levofloxacin (LVFX, 250 mg) (HER・LVFX for 2 months plus HR for 10 months), considering her medical history of liver cirrhosis and diabetes mellitus, because of the possibility of primary nasopharyngeal TB.

Although the posterior walls of the nasopharynx were still covered with a yellowish discharge after starting treatment, right vocal cord paralysis and right dysphagia developed on day 101.

CT revealed a diffuse enhancing mucosal irregularity in the right nasopharynx (Figure 1(c)) and bony erosion of the external skull base (Figure 1(d)). Nasopharyngeal carcinoma was the most important differential diagnosis owing to the neurological findings and the bony erosion. We performed a deep tissue biopsy using a rigid nasal endoscope and cupped forceps under local anesthesia and confirmed a pus discharge from the right nasopharynx. Histopathological examination showed no malignant findings, and the bacteriological examination of the pus was negative. On day 127, the patient's headache and neurological findings disappeared, and the yellowish discharge of the right nasopharynx had decreased. After that, we performed the same tissue biopsy and drainage again. On day 217, improvement of the bony erosion of the external skull base was confirmed (Figures 1(e) and 1(f)). On day 233, the yellowish discharge disappeared completely.

## 3. Discussion

Mycobacteriosis is classified as TB and NTM disease. TB infection is still one of the deadliest communicable diseases noted globally, with an estimated 10.0 million people acquiring the disease in 2019 [3]. Extrapulmonary TB accounts for 15% of all newly diagnosed TB cases worldwide, of which 10%–35% manifest in the head and neck region [4]. The most common head and neck TB sites are cervical lymph nodes (87.9%) and larynx (8.7%), but the involvement of other head and neck regions is rare (3.4%) [5]. Nasopharyngeal TB accounts for less than 1% of the head and neck TB cases [6, 7]. Nasopharyngeal TB can develop in a healthy patient without any underlying disease or history of tuberculosis contact or a compromised immune system [1]. The nasopharyngeal symptoms often include ear, nose, and other problems such as headaches. On nasopharyngoscopic examination, various nasopharyngeal findings have been reported, including normal appearance, irregular mucosa, ulcerative lesion, mass, bulging or swelling, a white patch over the nasopharyngeal area, and lymphoid hyperplasia. The standard treatment regimen is either a triple combination, such as HER for 9–18 months, or quadritherapy, such as HER plus pyrazinamide for 9 months [1].

On the other hand, the incidence of NTM disease is increasing worldwide [8]. Underlying health conditions pose a significant risk for NTM diseases [9]. In addition to pulmonary involvement, NTM frequently affects the skin, lymphatic tissues, and soft tissues [9]. The optimal treatment regimens and durations have not been established for most NTM species, as are surgical interventions [8].

The most important differential diagnosis in the present case was nasopharyngeal carcinoma, in addition to various diseases such as fungal infection (aspergillosis and mucormycosis), granulomatous inflammation (sarcoidosis, leprosy, syphilis, and midline lethal granuloma), and autoimmune disease (polyarteritis nodosa, eosinophilic granulomatosis with polyangitis, and granulomatosis with polyangitis) [4]. In the present case, the ulcerative mass covered with a thick yellowish discharge was typical of nasopharyngeal TB [2, 8]. The T-SPOT.TB test suggested an active TB infection with positive ESAT-6 and CFP-10, which are TB antigens [10]. Histopathological examination showed caseous necrosis in granulomas, epithelioid cell granulomas with Langhans giant cells, and the positive Ziehl–Neelsen staining of acid-fast bacilli without any malignant findings. Based on these findings, we eventually diagnosed the case as nasopharyngeal mycobacteriosis and administered treatment appropriate for nasopharyngeal TB.

The most characteristic feature of the present case was bony erosion of the skull base, which appeared and alleviated during the treatment course (Figure 1). Srivanitchapoom et al. reviewed nasopharyngeal TB and found that the lesions are usually confined to the nasopharyngeal area without invading the surrounding bony structures [1]. Cai et al. evaluated the CT and magnetic resonance imaging features of 36 nasopharyngeal TB cases and found no adjacent muscle invasion or bone destruction [2]. We could not retrieve any reports on primary nasopharyngeal TB or NTM with bone destruction in our literature search. However, bone destruction can be caused by progressed bone infection, and bone resorption-predominant destruction is commonly observed in osteoarticular TB. Izawa [11] investigated the mechanism of bone destruction in spinal TB by immunohistochemical analysis and suggested that the activation of the RANK-RANKL pathway resulted in bone destruction.

In the present case, antituberculosis medication alone was insufficient treatment; thus, the infection spreaded to the parapharyngeal space, formed an abscess, and resulted in glossopharyngeal and vagus nerve paralysis (Figure 1(c)). Bacterial abscesses, not just mycobacteriosis, in the parapharyngeal area can cause such cranial nerve palsies [12]. The progression of nasopharyngeal carcinoma can certainly result in cranial nerve palsy, which most commonly involves the trigeminal and abducens nerves and glossopharyngeal, vagus, and accessory nerves, when the invasion reaches the jugular foramen [13]. In the present case, differentiation from carcinoma was also important in terms of neurological findings.

Although a drug susceptibility test could not be performed, we presumed the causative bacteria as a drug-resistant TB or an NTM based on the clinical course and the patient's medical history. In such cases, the potential benefits of the surgical procedure as an adjunctive treatment for drug-resistant mycobacteriosis have been suggested [8, 14]. In the present case, although biopsy was the primary aim, drainage had led to a successful outcome.

This report describes a case of primary nasopharyngeal mycobacteriosis with bony erosion of the external skull base. Differential diagnosis considering both carcinoma and mycobacteriosis should be undertaken, even when CT shows a diffuse enhancing mucosal irregularity with bone destruction in the nasopharynx. Surgical intervention should also be considered as an adjunctive treatment for drug-resistant mycobacteriosis.
